# Laparoscopic resection for pedunculated focal nodular hyperplasia of the liver during pregnancy

**DOI:** 10.1093/omcr/omad054

**Published:** 2023-06-26

**Authors:** Ai Akaguma, Takamichi Ishii, Yoichiro Uchida, Yoshitsugu Chigusa, Yusuke Ueda, Masaki Mandai, Haruta Mogami

**Affiliations:** Department of Gynecology and Obstetrics, Graduate School of Medicine, Kyoto University, Kyoto, Japan; Department of Surgery, Graduate School of Medicine, Kyoto University, Kyoto, Japan; Department of Surgery, Graduate School of Medicine, Kyoto University, Kyoto, Japan; Department of Gynecology and Obstetrics, Graduate School of Medicine, Kyoto University, Kyoto, Japan; Department of Gynecology and Obstetrics, Graduate School of Medicine, Kyoto University, Kyoto, Japan; Department of Gynecology and Obstetrics, Graduate School of Medicine, Kyoto University, Kyoto, Japan; Department of Gynecology and Obstetrics, Graduate School of Medicine, Kyoto University, Kyoto, Japan

## Abstract

Focal nodular hyperplasia (FNH) is the second most common intrahepatic benign mass lesion; however, extremely rarely, FNH grows in an exophytic manner. It is unclear whether pedunculated FNH can be managed in the same way as intrahepatic FNH. A 35-year-old female presented with right upper quadrant pain, and dynamic enhanced computed tomography revealed an exophytic hyperdense mass lesion originating from the liver, suggesting a pedunculated FNH. Shortly thereafter, she conceived. Since there was a history of acute abdomen, as well as the possibility of torsion of the mass or sudden massive bleeding during pregnancy, laparoscopic resection was performed at 17 weeks of gestation. Her postoperative and pregnancy course was uneventful, and she delivered a baby by cesarean section at 41 weeks of gestation. Our case suggests that pedunculated FNH, unlike typical intrahepatic FNH, may be better managed by laparoscopic surgery during pregnancy, resulting in favorable maternal and fetal outcomes.

## INTRODUCTION

Pregnancies complicated with liver tumors or nodules, whether benign or malignant, are very rare, and thus, they present with challenges in diagnosis, management and treatment during pregnancy [1]. Focal nodular hyperplasia (FNH) is the second most common benign hepatic mass lesion and is characterized by a hyperplastic response to increased blood flow associated with intrahepatic vascular malformation [2]. In most cases, FNH is found asymptomatic as an intrahepatic nodule, and conservative management is recommended as a first-line approach. However, in extremely rare cases, FNH grows in an exophytic manner, which is called pedunculated FNH. It is uncertain whether pedunculated FNH can be managed similarly to intrahepatic FNH since the characteristics of pedunculated FNH, including the risks of rupture, hemorrhage and torsion remain unknown. The challenges increase when a pedunculated FNH is identified during pregnancy. We herein describe the first case of pedunculated FNH that was successfully treated with laparoscopic resection during pregnancy.

### Case presentation

**Figure 1:**
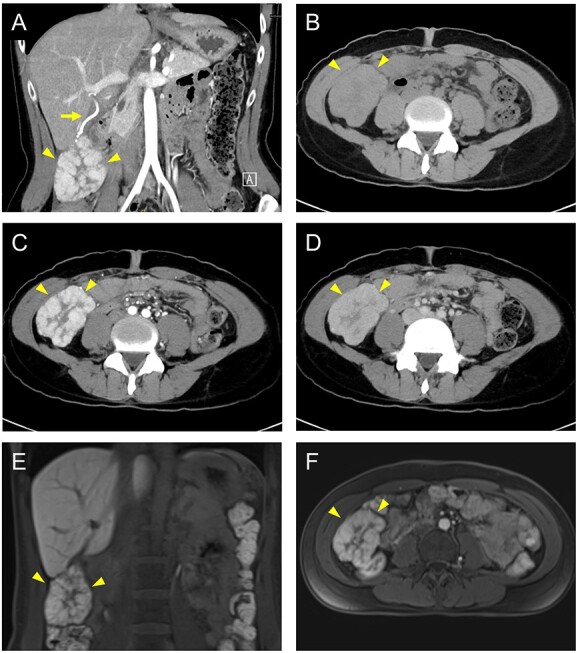
Findings of dynamic contrast-enhanced CT (**A**–**D**) and dynamic magnetic resonance imaging (**E** and **F**). (A) Coronal image demonstrates an exophytic hyperdense mass (arrowheads) and its feeder artery (arrow). (B) Native examination shows an isodense mass (arrowheads). (C) The multilobular mass is hyperdense and enhanced with a central stellate scar at the arterial phase (arrowheads). (D) The mass is still enhanced at the portal venous phase (arrowheads). On coronal (E) and axial (F) fat-saturated contrast-enhanced T1-weighted images, the mass demonstrates strong uptake of contrast agent at the early phase (arrowheads).

A 35-year-old female with a 15-year history of oral contraceptive use presented with right upper quadrant pain. A 7 cm mass lesion adjacent to the inferior border of the liver was detected by transabdominal ultrasound. Dynamic enhanced computed tomography (CT) revealed an exophytic hyperdense lesion originating from segment VI of the liver and its feeder artery (Fig. 1A). The mass was isodense at native examination, highly hyperdense with a central stellate scar at the arterial phase and still enhanced at the portal venous phase (Fig. 1B–D). Gadolinium ethoxybenzyl diethylenetriaminepentaacetic acid-enhanced dynamic magnetic resonance imaging also demonstrated strong uptake of the contrast agent at the early phase (Fig. 1E and F). These findings were suggestive of pedunculated FNH or hepatocellular adenoma. Given the symptomatic nature of this case, surgical resection was the treatment of choice, and oral contraceptives were ceased.

**Figure 2:**
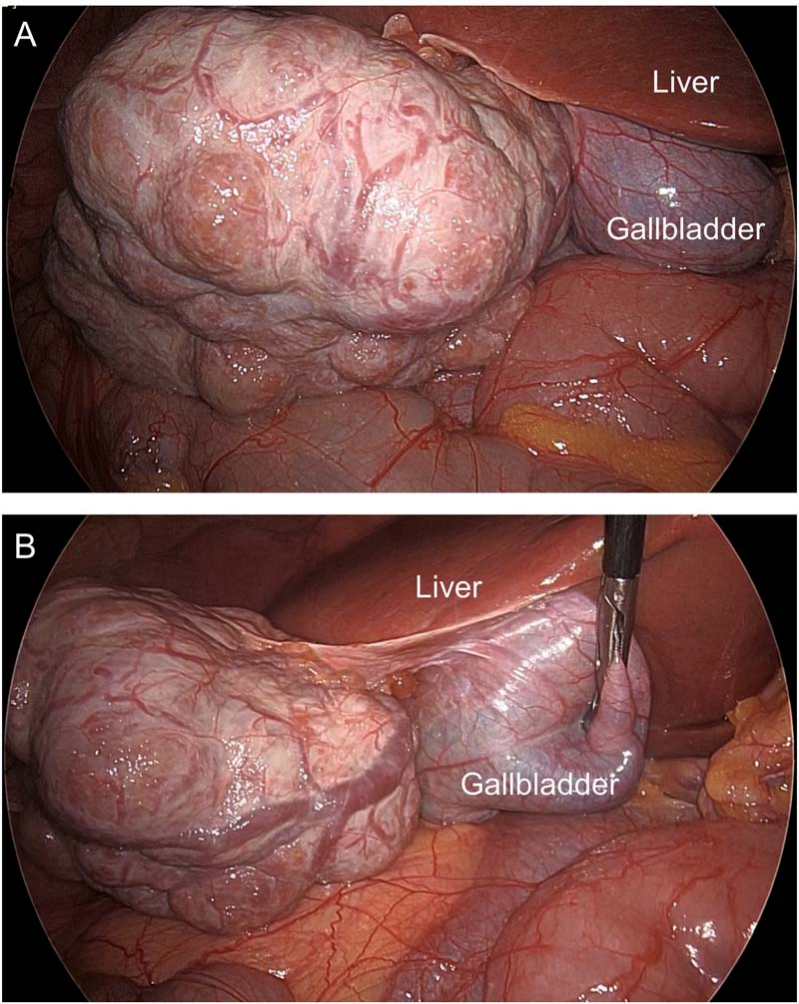
Intraoperative images. (**A**) A lumpy mass with numerous blood vessels on its surface is located near the lower margin of the liver. (**B**) The mass projects from the liver, and the blood vessels on the surface are overswelling.

**Figure 3:**
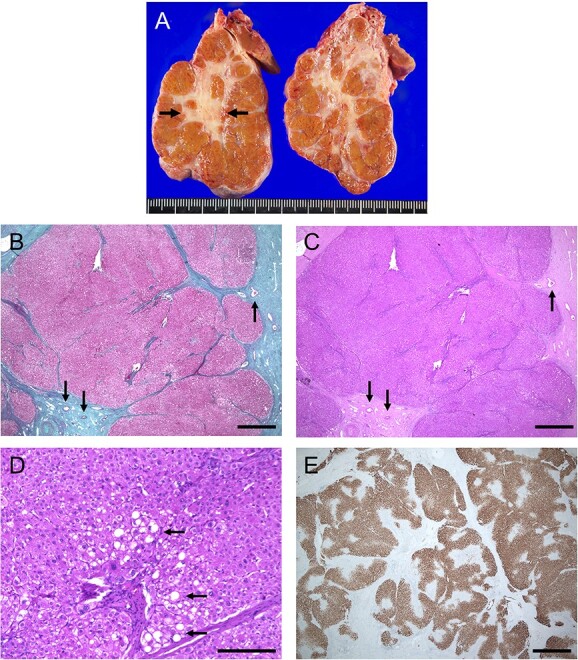
Pathological findings of resected specimens. (**A**) Gross examination shows a multinodular lesion with a central fibrous scar (arrows). (**B**) Masson trichrome staining and (**C**) hematoxylin and eosin staining show multiple islet-like nodules separated by a thick fibrous band. Fibrous septa contain blood vessels with thick walls (arrows) (Bar 1000 μm, ×20). (**D**) The cells that comprise the nodule resemble normal hepatocytes and have mildly enlarged nuclei. Partial fatty changes were detected (arrows) (hematoxylin and eosin stain, bar 200 μm, ×100). (**E**) Immunohistochemistry of glutamine synthetase shows a map-like pattern (Bar 1000 μm, ×20).

Then, she conceived and was referred to our hospital at 12 weeks of gestation. Since there was a history of acute abdomen, as well as the possibility of torsion of the mass or sudden massive bleeding during pregnancy, which could be life-threatening for both the mother and baby, the decision was made to perform surgery. At 17 weeks and 6 days of gestation, laparoscopic resection was successfully performed. During surgery, a lumpy mass with numerous overswelling vessels on the surface was observed projecting from the lower margin of the liver (Fig. 2A and B). Macroscopic examination of the resected specimen, measuring 7 × 7 × 5 cm, revealed a multinodular mass with a central fibrous scar (Fig. 3A). Microscopic examination showed multiple nodules separated by a thick fibrous band, and fibrous septa contained blood vessels with thick walls (Fig. 3B and C). The cells that comprised the nodule resembled normal hepatocytes and had mildly enlarged nuclei. Partial fatty changes were detected (Fig. 3D). Immunohistochemistry of glutamine synthetase showed a map-like pattern (Fig. 3E), leading to a diagnosis of FNH. Her postoperative and pregnancy course was preferable, and she delivered a female baby weighing 4270 g with Apgar scores of 8 and 9 at 41 weeks and 3 days of gestation, by cesarean section due to arrest of labor.

## DISCUSSION

FNH has an estimated incidence of 0.3–3% [1, 3] and occurs more frequently in women between the ages of 20 and 50 with a male-to-female ratio of 1:8 [3]. It was once reported that the majority of patients with FNH were oral contraceptive users [4]. These epidemiologic observations might suggest the association of sex hormones or oral contraceptives in FNH. However, the involvement of long-term use of oral contraceptives, which was also detected in the present case, in the development of FNH remains controversial, with some studies supporting it and others not [5, 6]. Generally, the risk of spontaneous rupture, acute bleeding or malignant transformation in FNH is very low. Therefore, as long as there are no symptoms or it is sufficiently distinguishable from other diseases, including hepatocellular adenoma or hepatocellular carcinoma, invasive treatments such as surgery and embolization are not performed, and conservative follow-up is recommended [7, 8].

Pedunculated FNH is an exceptional type of FNH and is an extremely rare entity. Only 13 cases of pedunculated FMH have been reported thus far (Table 1). All patients, including our patient, were female with a median age of 27.5 (range 3–48) years and a median mass size of 5.8 (range 2.9–19.5) cm. Notably, 11 of the 14 patients (79%) presented with some symptoms, and surgical treatment was chosen in 13 of the 14 cases (93%). These facts reflect the characteristics of pedunculated FNH very well. Presumably, unlike typical intrahepatic FNH, pedunculated FNH is more prone to various symptoms, such as abdominal pain or bloating, since it grows while pressing on other organs. Thus, it should be recognized that pedunculated FNH is more likely to require surgical treatment than typical FNH, even if it is asymptomatic when detected.

**Table 1:** Cases of pedunculated FNH reported in the literature

Author	Year	Age	Sex	OC (duration)	Size (cm)	Symptom	Treatment	PMID
Sawhney S	1992	12	F	No	N.D.	None	Open surgery	1508599
Bader TR	2001	N.D.	F	N.D.	4.5	Upper abdominal pain	Surgery	11672619
Byrnes V	2004	30	F	No	18	Right upper quadrant pain	Open surgery	15118579
Wasif N	2008	48	F	No	3.2	Right upper quadrant pain	Laparoscopic surgery	19062669
Khan MR	2011	29	F	No	19.5	Pain in the right quadrant	Open surgery	21269944
Terada T	2012	26	F	No	5	None	Laparoscopic surgery	22686860
Eris C	2013	23	F	Yes (1 year)	7.8	Abdominal pain, vomiting	Open surgery	23792480
Reddy K	2015	35	F	N.D.	2.9	Abdominal pain	Laparoscopic surgery	25527059
Badea R	2015	29	F	Yes (3 years)	8	Pain in the right hypochondrium	Laparoscopic surgery	26578496
Zeina AR	2016	25	F	N.D.	4.8	Epigastric pain	Observation	27740528
Koolwal J	2018	3	F	No	6.5	Abdominal distention	Open surgery	29686569
Navarini D	2020	26	F	N.D.	13	Acute abdominal pain, torsion	Open surgery	32527652
Ben Ismail I	2021	38	F	Yes (3 years)	4.5	None	Open surgery	34136232
Ours	2022	35	F	Yes (15 years)	7	Acute abdominal pain	Laparoscopic surgery	

OC: oral contraceptive, F: female, N.D.: not described.

PMID: PubMed ID

The biological behavior of FNH during pregnancy is debatable, even though the link between FNH and estrogen exposure has been speculated. In most cases, the size of FNH was stable during pregnancy, whereas there have been some cases of FNH growing in pregnancy and reports of FNH shrinkage after childbirth [9–11]. Chandrasegaram *et al*. reported that estrogen receptor nuclear staining was strongly positive in two out of 13 cases (15%) of FNH [11], and the presence or absence of expression of these receptors might influence the behavior of FNH during pregnancy.

In the present case, we decided that the hepatic mass lesion should be surgically removed early in the second trimester of pregnancy. First, the patient had previously experienced severe abdominal pain, even though it resolved spontaneously, and there was the possibility that the symptom would recur. Second, since the nodule was extremely hypervascular, there was a significant risk of rupture and heavy bleeding if the nodule came into contact with the enlarged uterus. Third, for similar reasons, torsion of the nodule could occur in pregnancy. Fourth, although imaging tests led us to suspect pedunculated FNH, hepatic adenoma could not be completely ruled out, and there is a considerable risk of rupture during pregnancy. Collectively, we determined that the benefits of surgical treatment in the present case greatly outweigh the potential risks that might arise from the wait-and-see strategy.

In conclusion, pedunculated FNH may require laparoscopic surgical treatment during pregnancy, unlike typical intrahepatic FHN, to achieve a definitive diagnosis and ensure favorable maternal and fetal outcomes.

## Data Availability

The datasets analyzed during the current study are available from the corresponding author on reasonable request.
